# Effect of pH on the Supramolecular Structure of *Helicobacter pylori* Urease by Molecular Dynamics Simulations

**DOI:** 10.3390/polym12112713

**Published:** 2020-11-17

**Authors:** Haruna L. Barazorda-Ccahuana, Badhin Gómez, Francesc Mas, Sergio Madurga

**Affiliations:** 1 Materials Science and Physical Chemistry Department & Research Institute of Theoretical and Computational Chemistry (IQTCUB), University of Barcelona, C/Martí i Franquès, 08028 Barcelona, Spain; hbarazorda@gmail.com (H.L.B.-C.); fmas@ub.edu (F.M.); 2 Centro de Investigación en Ingeniería Molecular–CIIM, Vicerrectorado de Investigación, Universidad Católica de Santa María, Urb. San José s/n Umacollo, Arequipa 04013, Peru; bgomez@ucsm.edu.pe

**Keywords:** molecular dynamics, semi-grand canonical, Monte Carlo, urease

## Abstract

The effect of pH on the supramolecular structure of *Helicobacter pylori* urease was studied by means of molecular dynamics simulations at seven different pHs. Appropriate urease charge distributions were calculated using a semi-grand canonical Monte Carlo (SGCMC) procedure that assigns each residue’s charge state depending on the assigned individual p$K_{a}$ obtained by PROPKA. The effect of pH on protein stability has been analyzed through root-mean-square deviation (RMSD), radius of gyration (RG), solvent-accessible surface area (SASA), hydrogen bonds (HB) and salt bridges (SB). Urease catalyses the hydrolysis of urea in 12 active sites that are covered by mobile regions that act like flaps. The mobility of these flaps is increased at acidic pHs. However, extreme acidic conditions cause urease to have the least number of stabilizing interactions. This initiates the process of denaturalization, wherein the four ${\left(\alpha \beta \right)}_{3}$ subunits of the global structure ((αβ)${}_{3}$)${}_{4}$ of urease start to separate.

## 1. Introduction

*Helicobacter pylori* (HP), a Gram-negative helical bacterium, infects about half of the world’s population, inducing gastritis, peptic ulcers and increased risk of gastric cancer [1,2]. Interestingly, HP is highly adapted to the medium found in the stomach. First, HP stays in the lumen with a pH of about 2.0, and then colonizes the mucus layer which has a pH that ranges from 4.5 to 6.5 [3]. HP is a spiral-shaped Gram-negative bacterium, microaerophilic and highly motile due to the presence of 4–8 flagella [4]. Cheng-Yen Kao et al. described four fundamental steps for colonization, which consist of survival, motility, adhesion and toxicity [5]. One of the key characteristics that makes HP to survive at acidic conditions is the generation of large quantities of the urease enzyme.

Urease is an enzyme that catalyses the hydrolysis of urea to ammonia and carbamate. Carbamate further decomposes to ammonia and carbonic acid. The protonation of ammonia and dissociation of carbonic acid causes an increase in the pH, allowing HP to survive in the pH conditions of the stomach. Thus, this important role in the survival of HP bacteria makes urease an excellent target for inhibitors.

Some studies have been performed to elucidate the mechanism of action of the enzyme and to identify suitable inhibitors for HP urease using quantum mechanical (QM), molecular dynamics (MD) and docking methodologies. QM/MM methodology was applied to study the mechanism of inhibition of hydroxamic acids acting on *Helicobacter pylori* urease [6]. Mechanistic pathways were also explored in combination with MD simulations based on hydrogen-bonding network of the catalytic center [7]. Molecular dynamics (MD) studies reveal the mobility of regions called flaps that open and close the access to the active center of the urease [8,9]. Docking calculations have been performed to identify potential inhibitors of urease that could interact with the active center [10] or could lock the flexible loop in open conformation [11].

The first crystallized urease was jack bean urease [12]. It was observed that urease has a complex structure requiring nickel ions for its activity. Nam Chul-ha et al. analyzed the supramolecular assembly of HP urease, identifying two functional subunits named α and β of 26.5 and 61.7 kDa, respectively [2]. The α and β subunits constitute a heterodimeric unit. Then, three heterodimeric units of αβ ((αβ)${}_{3}$) form the tetrahedral complex ((αβ)${}_{3}$)${}_{4}$ which turns out to be the structure of urease [2]. Additionally, the macromolecular complex of urease presents an internal hollow with a diameter of 1.3 nm. HP urease has 12 active sites, each being coordinated with two Ni${}^{2+}$ ions. It is one of the most important regions of the enzyme that could protect it from the acidic environment by urea hydrolysis. The active site is covered by a protein region, denominated “flap”, having high flexibility that could change from closed to open state in order to promote the access of urea at the dinickel center [9,11].

For a long time, the pH dependence in structural protein stability has been considered. Decreased stability at more acidic pH is explained by the increase of unfavorable electrostatic interactions due to the high increase of distributed positive charges, and the contrary occurs with negative charges at a highly basic pHs [13]. Therefore, the pKa values of ionizable groups in proteins play essential roles in their stability and functions [14].

The optimum pH of urease is known to be approximately 7.5, and below a pH of 4.5 the enzyme has been reported to be inactive [15]. However, the effect of pH on the mechanism of urease is still not known. For this reason, in this work we determined the different protonation states at seven pHs by semi-grand canonical Monte Carlo (SGCMC) [16,17,18,19] and the behavior of each system through molecular dynamic simulations.

## 2. Computational Details

### 2.1. Multimeric Protein Preparation

Urease is a protein with a dodecameric ((αβ)${}_{3}$)${}_{4}$ architecture with spherical assembly. The fundamental unit is a heterodimer with two chains labeled α and β. The crystal structure obtained from the Protein Data Bank (PDB) with the access code 1E9Z was used as initial structure. Urease has 12 active sites located on the β chains. The residues of this chain that participate in the active site are a set of four histidines (His136, His138, His248, and His274), a carbamylated lysine (KCX219), an aspartic acid (Asp362), two nickel ions (Ni${}^{2+}$) and a hydroxide ion. The non-natural carbamylated lysine was parameterized using the atom types and parameters corresponding to the similar fragments of lysine and aspartic acid. Force field parameters of the modified carbamylated lysine amino acid used in the Gromacs topology file are given in the SI.

**Figure 1:**
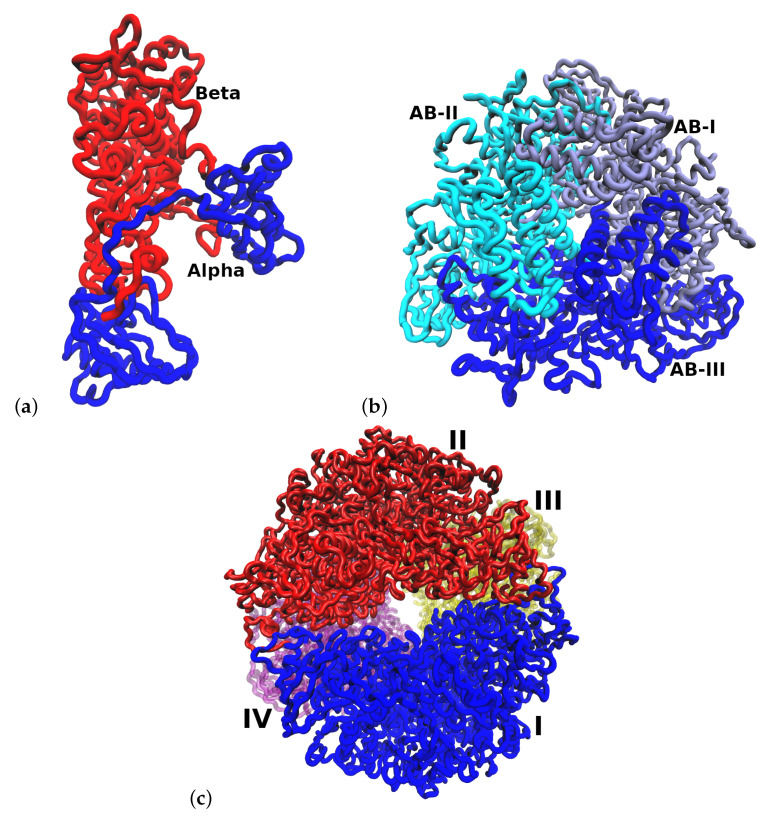
Structural arrangement of HP urease at pH 7.5. (**a**) Heterodimer (αβ). (**b**) Arrangement of one ${\left(\alpha \beta \right)}_{3}$ subunit. (**c**) Entire urease structure where each ${\left(\alpha \beta \right)}_{3}$ is shown in a different color and labeled with I, II, III and IV.

HP urease has an interesting structural arrangement with different levels of chain assembly. Figure 1 shows three different levels of organization of urease protein. The fundamental unit is a heterodimer (αβ) which interacts with two other heterodimers of the ${\left(\alpha \beta \right)}_{3}$ assembly, and finally, this assembly interacts with three other ${\left(\alpha \beta \right)}_{3}$ assemblies, completing the full structure ${\left({\left(\alpha \beta \right)}_{3}\right)}_{4}$ [2].

### 2.2. Protonation States of Urease at Different pHs

In order to determine the charge state of each titrable residue of urease, and to determine how it changes with the simulated pH, it is required to have the p$K_{a}$ values of all titrable residues of the protein. The urease structure was evaluated using PROPKA [20], which predicts the p$K_{a}$ values of ionizable groups based on the 3D structure. PROPKA uses the terms indicated in Equation (1) to estimate the p$K_{a}$ value of each titrable residue:

(1) $$pK_{a}=pK_{\mathrm{model}}+\Delta pK_{\mathrm{DS}}+\Delta pK_{\mathrm{HB}}+\Delta pK_{\mathrm{CC}}$$

where p$K_{\mathrm{model}}$ is the p$K_{a}$ of the ionizable groups in an ideal system; p$K_{\mathrm{DS}}$ takes into account the penalty of desolvation; p$K_{\mathrm{HB}}$ considers the effect of hydrogen bonds of the side chain and backbone; and p$K_{\mathrm{CC}}$ takes into account the effect of the interaction of the charge groups.

We have designed a homemade *c* program to calculate the different protonation states of Asp, Glu, Arg, Lys and His residues, and the C- and N- termini for seven pHs (2, 3, 4, 5, 6, 7 and 7.5) using the semi-grand canonical Monte Carlo (SGCMC) ensemble [16,17,18,19,21]. A characteristic feature of semi-grand canonical Monte Carlo (SGCMC) simulations is that the total charge is not constant but it fluctuates as a natural result of the dynamic proton binding/unbinding. The required inputs of the program are the pH, number of trial protonation/deprotonation steps and the list of acid/base constants in the format given by the summary of Propka. Initially, all protonable residues are assigned the protonated stated. The system arrives quickly to the equilibrium, as the p$K_{a}$ values are considered to be independent, being unaffected by the protonated state of neighbor residues. The histidines that coordinate the Ni2+ ions are fixed to be in the appropriate deprotonated state to correctly coordinate the metal ions. The program calculates the total charge, the distribution of protonated/desprotonated forms for each residue and generates a output file with the format that the *pdb2gmx* command of Gromacs requires for the interactive selection of charged states.

The free energy associated with the protonation/deprotonation of each titrable group is calculated using Equation (2) [16,17].

(2) $$\Delta G=\pm k_{B}T(ln\left(10\right)\left(\mathrm{pH}-pK_{a,i}\right)$$

The positive sign of expression of Equation (2) is used for protonation and the negative sign for deprotonation. $K_{a,i}$ is the constant of the acid/base equilibrium corresponding to each protonable *i* residue of urease determined by PROPKA. A total of 30,000 steps for protonation and deprotonation trials for residues of urease were performed with the SGCMC simulation program at each pH. As the protonation states are obtained from pKa values calculated with PROPKA using the crystal structure of urease, these protonation states will correspond correctly to the assigned pHs if the global structure of the urease does not change significantly. Furthermore, the effect of the explicit Na or Cl ions included in the simulations to neutralize the systems is not taken into account in the selection of the protonated or deprotonated states of the titratable residues.

### 2.3. Molecular Dynamic Simulations

All molecular dynamics simulations were performed with Gromacs (Groningen Machine for Chemical Simulations) version 2019.2 software [22,23] using the OPLS-AA force field. Seven different initial structures of urease were prepared while attending to the different charge states of the titrable amino acids obtained from the SGCMC calculations corresponding to each simulated pH. In each case, the urease structure was located in the center of a box with dimensions about 18 nm${}^{3}$ having a minimum separation of 1.5 nm to the box edges. Distances of the Ni${}^{2+}$ with the coordinated residues of the active site were restrained at the values of the crystal structure because there are not available bonding parameters for nickel metal ions in the used force field. The protein was solvated with TIP4P [24] waters and the system was neutralized with the addition of the appropriate quantity of Na${}^{+}$ or Cl${}^{-}$ (Table S1). It is worth knowing that the salt concentration of ions required to neutralize the system is higher than the normal physiological salt concentrations (0.154 M) for the more acidic pHs, being, for example, 0.4 and 0.35 M for pHs 3 and 4, respectively. However, although the survival of *Helicobacter pylori* has been reported to be optimal at physiological salt concentrations, it survives also at high concentrations, as the simulated in this study [25].

Initially, the systems were relaxed with an energy minimization using the steepest descent algorithm with 20,000 steps while applying 0.001 nm as a maximum step size. Periodic boundary conditions were applied in all directions. Short-range interactions of van der Waals (vdW) and electrostatic interactions were considered with a cut-off of 0.9 nm. Long range electrostatic interactions were considered with the particle-mesh Ewald (PME) [26] method.

Preparation of the systems was performed with MD in a canonical ensemble with V-rescale [27] thermostat (309.65 K) during 10 ns. Finally, an isobaric-isothermal ensemble with V-rescale thermostat (309.65 K) and Parrinello–Rahman [28] barostat (1.0 bar) were performed for a simulation length of 100 ns. Molecular visualization of the different systems was performed using Visual Molecular Dynamics version 1.9.4. [29] and Chimera UCSF version 1.12.0 [30]. All plot analysis were performed with Gnuplot version 5.3 software.

Simulations corresponding to pHs 2 and 7.5 were prolonged for 100 ns in order to check that the simulation length was appropriate for obtaining converged results. In Table S2 are the statistics of the poor and favored rotamers and of the Ramachandran outliers and favored angles after the NVT and NPT simulations. It can be seen that for all pH, the 10 ns NVT simulations improved the initial quality of the structure. Ramachandran outliers were reduced significantly, and residues in Ramachandran-favored regions achieved the stability value. Subsequently, the 100 ns NPT simulations were able to obtain a greater number of rotamers in favored regions. The results obtained from the 100 ns NPT continuation simulations indicate that longer simulations are not required to obtain equilibrated rotamers and Ramachandran angles.

## 3. Results and Discussion

### 3.1. Urease Charge as a Function of pH

**Figure 2:**
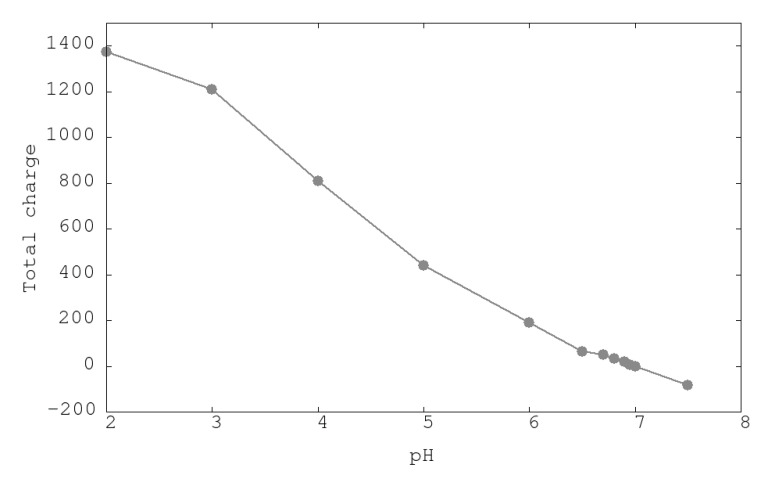
Total urease charge obtained from SGCMC simulations of titrable amino acids at different pHs.

The appropriate distribution of charged residues over the proteins will confer structural stability. This distribution is a function of the pH of the medium. In Figure 2, a monotonic decrease of the positive total charge of the urease from pH 2 until pH 7 can be seen. After this pH, the urease changes the total charge from a positive to a negative sign, indicating this point, 7.0, as the isoelectric point (IP) of urease. Experimental IPs of urease range from 5.4 [31] to 6.1 [32]. Differences with calculated IP could be attributed to precision of calculated intrinsic constants of titrable residues and/or the effects of condensed counterions in the internal cavity of the urease protein that could distort the deduced protein charge obtained by usual techniques.

In Table 1 the charge as a function of pH obtained from SGCMC simulations for the Asp, Glu, Arg, Lys and His titrable amino acids and for the entire structure of the urease can be seen. It can be seen that the greatest contributions to the variation of the charge are because of the Asp and Glu acid residues. From pH 2 to pH 7.5, the charges change from −7 and −33 to −575 and −653 for Asp and Glu, respectively. In contrast, the effect of the variation of the charge of Arg and Lys basic residues is very reduced as the average p$K_{a}$ of these residues (Table 2) is located outside of the studied pH range (average p$K_{a}$ of 12 and 10 for Arg and Lys, respectively). It is worth noting that the His residues show the wider distribution of p$K_{a}$ (4±3). This could be the result of a more variable environment for residues of this kind.

### 3.2. Structural Changes at Different pHs: RMSD, RG and SASA

**Figure 3:**
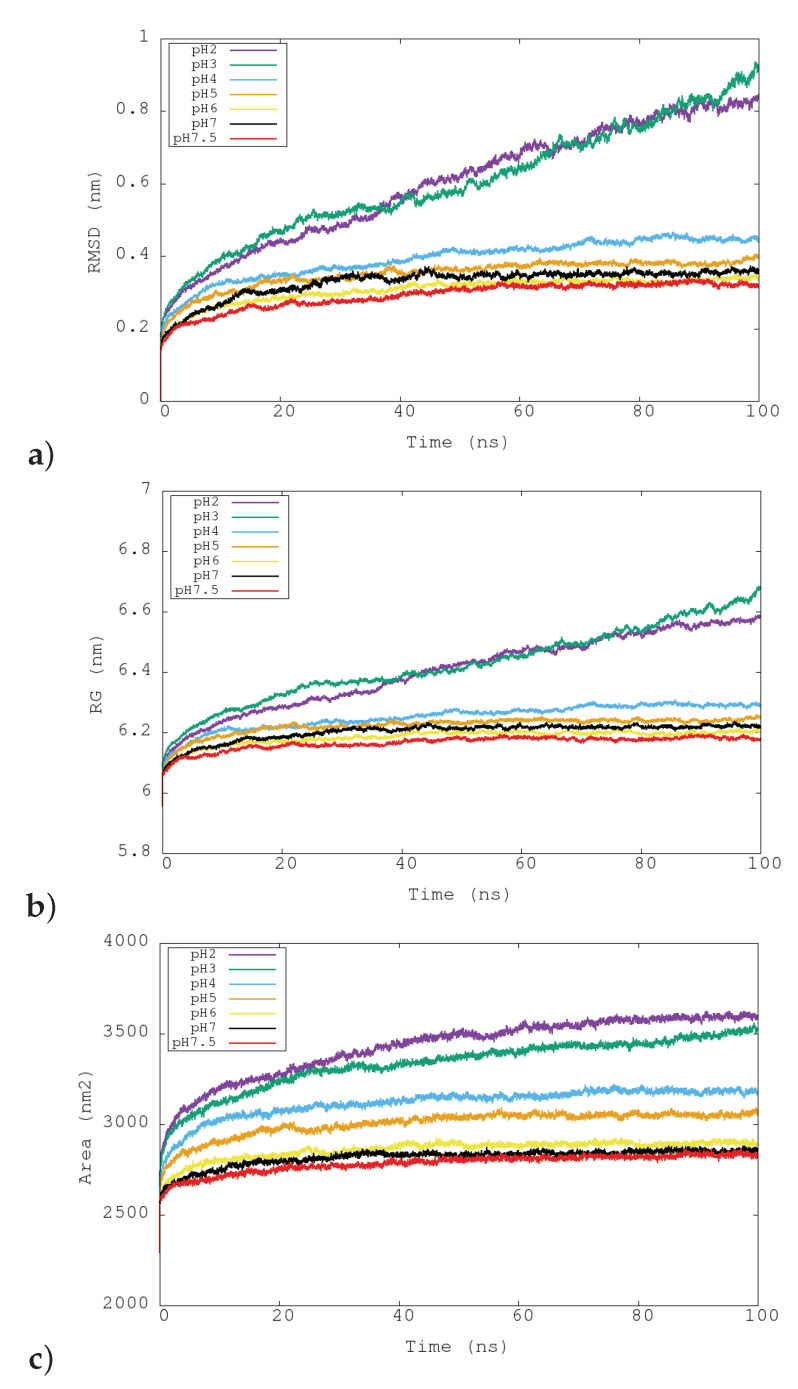
Representation of the temporal evolution of structural properties of urease in the molecular dynamics simulations at pH 2, 3, 4, 5, 6, 7 and 7.5. (**a**) Root-mean-square deviation (RMSD) for the total *Helicobacter pylori* urease. (**b**) Radius of gyration (RG). (**c**) Solvent accesible surface area (SASA).

The temporal evolution of the root-mean-square deviation (RMSD) of the backbone atoms of the entire structure of urease is shown in Figure 3a. Two patterns can be distinguished, depending on the simulated pH conditions. Simulations from pHs of 4 to 7.5 show a plateau in the RMSD starting approximately around 40–50 ns. However, simulations at pHs 2 and 3 show a linear increase of RMSD with time after 20 ns. The fact that the RMSD profile does not arrive at a constant value with more time indicates that the urease structure is not stable at the two more acidic pHs. To better quantify this effect, the average RMSD values through the last 20 ns were computed (Table 3). The average RMSD values show a differentiated behavior for the two more acidic pHs (about 0.8 nm) with respect to the other cases (0.32–0.45 nm). It has to be noted that pH 4 and pH 5 have greater RMSD values than the more basic pHs, indicating more structural flexibility of the protein for these intermediate pHs with respect to the neutral cases.

The effect of structural distortion could be analyzed for the different simulated pHs by computing the radius of gyration (RG) Figure 3b. As in the analysis of global RMSD, two patterns could be distinguished depending on the simulated pH conditions. pHs 2 and 3 showed a linear increase of RG, whereas pHs 4 to 7.5 show a plateau. Thus, the two more acidic conditions presented a structure that is increasing in size during time. The other conditions showed again a stable structure which is structurally more compact. In the last 20 ns, the RG values of the two more acidic conditions were about 0.4 nm greater that those corresponding to the other pH conditions (Table 3). Thus, the analysis of the radius of gyration of the entire structure of the ureases allows us to identify the increase of the size at acid conditions. In order to distinguish if this growth is performed on the external surface, and if the inner part of urease is more separated or more compact, two new calculations of radius of gyration were performed while selecting only the internal Glu505 (to analyze the size variation of the internal cavity) and the external Glu101, Glu116 and Glu181 (to analyze the size variation of the external surface). The obtained values indicate that both internal and external RG increase at acidic conditions in the same range (Table 3). Differences between pHs 2 and 3 seem to be attributable to statistical fluctuations. Thus, these values indicate that urease is increasing in size at the same time that the dimensions of its internal cavity increase.

**Figure 4:**
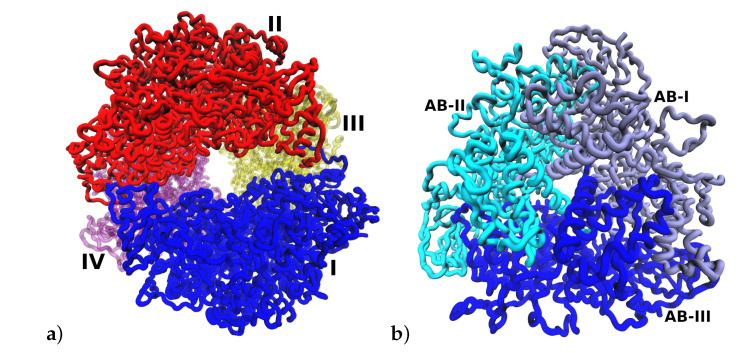
Representation of urease at pH 2. (**a**) ${\left({\left(\alpha \beta \right)}_{3}\right)}_{4}$ multimeric structure. Each ${\left(\alpha \beta \right)}_{3}$ is shown in a different color and indicated with I, II, III and IV. The separation of cluster I and cluster II increases at pH 2. (**b**) Similar structural arrangement for the ${\left(\alpha \beta \right)}_{3}$ subunit.

The variation of the solvent-accessible surface area (SASA) with time for the different pHs is shown in Figure 3c. The greatest SASA values were obtained for conditions of pH 2 and 3. This behavior is related to the observed increased of RG for these pHs. The more compact structures in terms of surface were obtained for the more basic pHs. The increase of size at acidic pHs can be also noticed in Figure 4a where an increase in the separation between I (blue) and II (red) ${\left(\alpha \beta \right)}_{3}$ subunits in the final frame of the simulation at pH 2 can be seen. This disaggregation phenomenon is in correspondence with mass spectrometry studies that observe that the dodecamer disassembles readily into ${\left(\alpha \beta \right)}_{3}$ subunits in solution and under controlled collision-induced dissociation in the gas phase [33].

### 3.3. Analysis of Stability at Different pHs: HB and SB

The number of the total intramolecular hydrogen bonds is a measure of the internal stability of the protein. Gromacs tools were used to analyze the hydrogen bonds considering all possible donor and acceptor atoms with a geometric criterion of a distance of less than 3.5 Å and an angle of $180^{\circ }\pm 30^{\circ }$.

**Figure 5:**
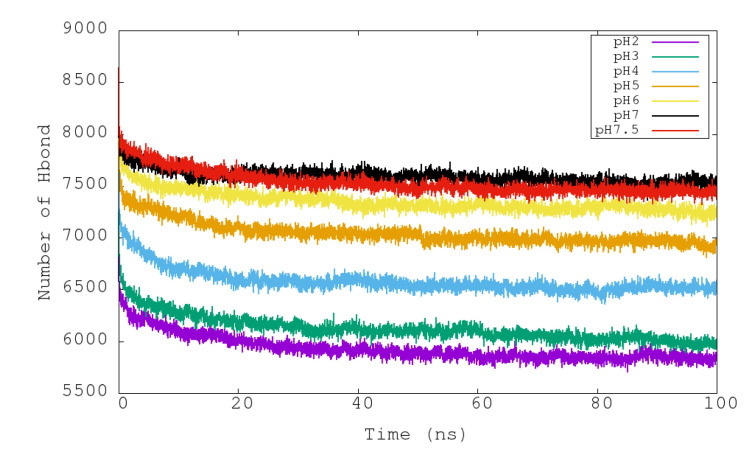
Representation of the temporal evolution of the number of total intramolecular hydrogen bonds of urease in molecular dynamics simulations at pH 2, 3, 4, 5, 6, 7 and 7.5.

In Figure 5, the variations of the intramolecular hydrogen bonds of urease with respect to time at different pHs can be seen. In contrast to the observed pattern for RMSD and RG properties, the total number of hydrogen bonds shows a more gradual variation among the simulated pHs. Simulations at pH 7 and 7.5 presented the largest numbers of intramolecular hydrogen bonds (around 7500), and then they diminished progressively with lower pHs to arrive at about 5900 for pH 2 (Table 3). The reduced number of hydrogen bonds at more acidic conditions could explain the reduced structural stability and the increase in size of the urease with time.

**Figure 6:**
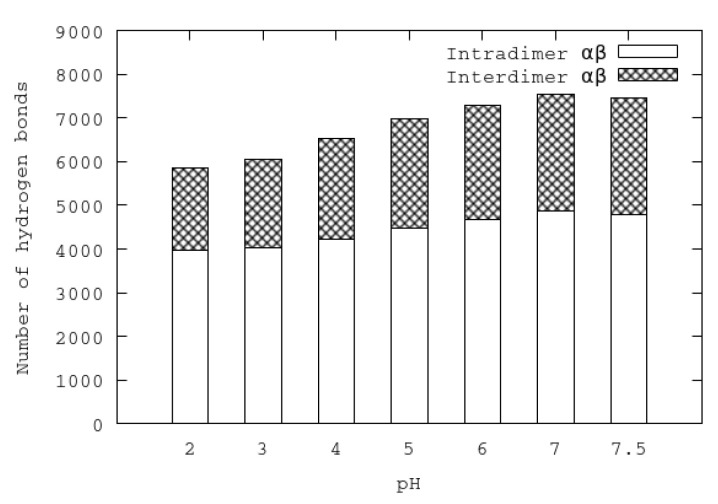
Numbers of average inter- and intradimer hydrogen bonds in urease at different pHs.

An important feature of urease is its tetrahedral arrangement of four subunits of (αβ)${}_{3}$. The interactions of (αβ) dimers were analyzed at different pHs by means of hydrogen bonds. Each subunit αβ establishes hydrogen bond interactions with two neighbor subunits αβ (of the same ${\left(\alpha \beta \right)}_{3})$, and to a lesser extent, with the other αβ subunit of another ${\left(\alpha \beta \right)}_{3}$ arrangement. All the hydrogen bond interactions established between atoms of different subunits are referred as interdimer hydrogen bonds. In contrast, all the hydrogen bonds established between atoms of the same dimer are referred as intradimer hydrogen bonds. In Figure 6, the average of the last 50 ns of the inter- and intradimer αβ hydrogen bonds for each pH is shown. It can be seen that the numbers of both inter- and intradimer αβ hydrogen bonds diminish at acidic pHs. The separation of ${\left(\alpha \beta \right)}_{3}$ subunits at acidic pHs could be attributed to the reduction of interdimer hydrogen bonds. On the other hand, the reduction of the number of intradimer hydrogen bonds could make the individual subunits more flexible and mobile.

Another property that provides information about the structural stability of urease is the number of established intramolecular salt bridges (SB). SB were calculated using the salt bridges plugin of VMD based on a cut-off distance of 3.2 Åbetween any oxygen atom of an acidic residue and nitrogen atom of a basic residue. It can be seen in Table 3 that the numbers of SB corresponding to the last frame of pHs 2 and 3 (96 and 131, respectively) are significantly lower than in the other pH conditions.

It is worth noting that the more acidic conditions that are shown to be less structurally stable correspond to the conditions where there is not experimental activity of the urease (Table 3). At pH 7.5, it is found to have the greatest experimental activity, and the simulation at this pH shows the lowest values of SASA and RG. It seems that the compactness of the protein is related to high enzymatic activities.

### 3.4. Mobile Flap at Different pHs

**Figure 7:**
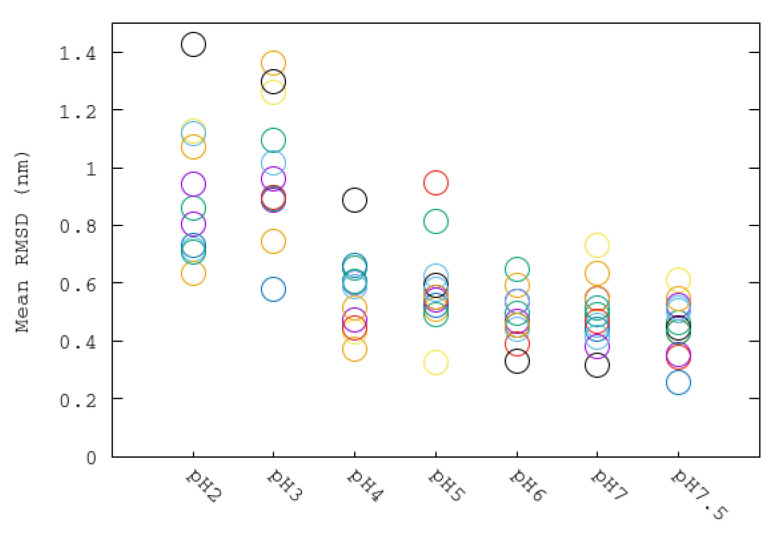
Average root-mean-square deviation (RMSD) values of backbone atoms of each flap at different pH simulations. Each flap is aligned with respect to the backbone atoms of its corresponding dimer. Each flap is displayed with a different color.

Urease has 12 binding pockets with two Ni${}^{+2}$ each. In the crystal structure of urease, it can be seen that these binding pockets are hidden by fragments composed of residues 304–347 of the beta monomers. These regions, named flaps, have been reported to be flexible and mobile, allowing the entrance of substrates to the binding pockets [9]. The average RMSD of the backbone atoms of each flap is represented as a function of the simulated pH in Figure 7. The temporal evolution of RMSD of the 12 flaps of urease enzyme at different pHs can be also seen in Figure S1. It can be seen that highest RMSD values were obtained for the most acid condition, indicating the greatest distortion with respect to the close state of the flaps. The lowest RMSD values are for those flaps simulated at pHs from 6 to 7.5, whereas the more acidic conditions correspond to states having more flaps in a more open state. Mean RMSD values and their corresponding dispersion are greater for the more acidic pHs, indicating a less stable urease structure. A similar pattern found for flap RMSD was found for the calculated RMSD values of dimers (Figure S2). pHs 2 and 3 have greater RMSD values and a greater dispersion among dimers, whereas RMSD values of dimers of pHs 4 to 7.5 have similar and more stables values.

**Figure 8:**
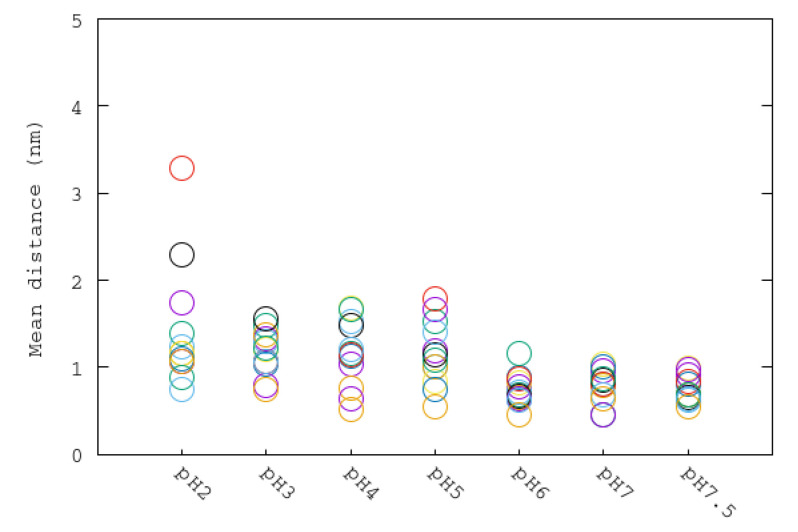
Average nickel–Cys321 distances of each subunit for all pH simulations. Same color code as Figure 7.

**Figure 9:**
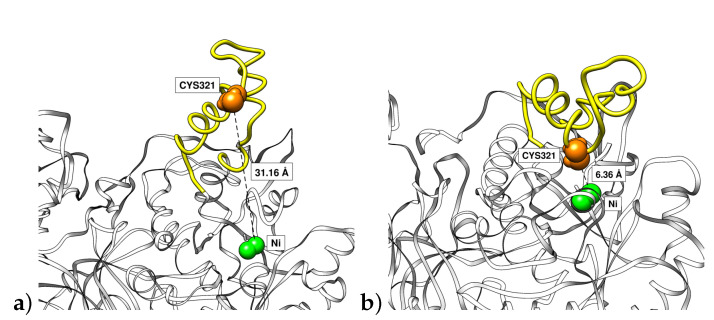
Final snapshots of one urease flap (yellow) in the simulations at (**a**) pH 2 in open conformation and (**b**) pH 7.5 in closed conformation. Ni${}^{+2}$ ions are represented in green. Distances of Ni${}^{+2}$ to Cys321 (orange) are also indicated.

In addition, the process of the opening of the flap could be analyzed by means of the distance of residue Cys321 to one of the Ni${}^{+2}$ ions (Figure 8). As Cys321 is a residue located in the central part of the flap, an increase of the distance with respect to the nickel ion of the binding pocket implies an opening of the flap. It can be seen that the fluctuation of these distances among the flaps has the general tendency to increase with the reduction of the pH. The greatest difference was observed for the more acidic condition having two flaps with the greatest average distances among all simulations. In Figure 9a we show the Cys321–Ni${}^{+2}$ distance corresponding to one of the flaps at pH 2 (3.1 nm) being significantly greater than thay corresponding to the flap at pH 7.5 (0.6 nm) in closed state (Figure 9b). This important fluctuation in conjunction with the results obtained from RMSD of flap residues indicates that the pH is an important factor that modulates the mobility of the flaps. It can be seen that the highest mobilities are obtained for the more acidic pHs.

The activity of urease could be related to the ability of reactants to access each of the 12 active centers. This access seems to be regulated by the region that acts as a flap. The effect of reduction of pH is to increase the mobility of this flap. Thus, a reduction of pH is expected to increment the protein’s activity. However, at extremely acidic pHs, the most important effect is the significant reduction of the stabilizing interactions (intermolecular and intramolecular non-bonded interactions). This important reduction of stabilizing interactions in combination with the absence of stabilization of the RMSD of the entire urease seems to indicate a denaturation process at very high pHs. However, at moderate acidic pHs, the increase of mobility of these flaps could facilitate urea’s access to the active site, increasing the pH in the environment of urease proteins in order to avoid their denaturation.

## 4. Conclusions

The effect of pH on the supramolecular structure of *Helicobacter pylori* urease has been studied with a set of molecular dynamics simulations. The different protonation states of titrable residues of urease have been determined by semi-grand canonical Monte Carlo (SGCMC) at different pHs.

The SGCMC procedure used to obtain the urease charge distribution seems to be an efficient method to generate different charge states to simulate proteins under a wide range of pH conditions [16,17,18,19]. This procedure assigns charges to monomers of the protein that are not identical but are statistically compatible with the simulated pH.

Molecular dynamics simulations performed until 100 ns allow one to understand the activity of urease in a wide range of pHs. At more acidic conditions represented with simulations at pH 2 and 3, the subunits ${\left(\alpha \beta \right)}_{3}$ start to separate. This would lead to the disaggregation of the protein at these extreme pH conditions. Protein stability could be also analyzed by means of inter- and intra-dimer hydrogen bonds and salt bridges. Extreme acidic conditions cause urease to have the least number of these stabilizing interactions. This behavior correlates well with the experimental activity of the urease observed only in acidic media greater than pH 5 [15,34].

The measured RMSD, RG and SASA properties and HB and SB analysis seem to be appropriate indicators to study protein stability at different pHs through molecular dynamics simulations.

## Figures and Tables

**Table 1:** Charge as a function of pH for titrable amino acids and for the entire structure of the urease.

System	ASP	GLU	ARG	LYS	HIS	Urease
pH2	−7	−33	360	792	237	1373
pH3	−73	−99	360	792	204	1208
pH4	−246	−281	360	791	162	810
pH5	−415	−464	360	789	144	438
pH6	−505	−566	360	778	98	189
pH7	−554	−627	360	757	37	−3
PH7.5	−575	−653	359	744	18	−83

**Table 2:** Number an average p$K_{a}$ values of titrable amino acids of urease.

AA	Number	p$K_{a}$
ASP	624	4.7 ± 1.6
GLU	696	4.6 ± 1.6
ARG	360	12.3 ± 1.5
LYS	792	10.0 ± 1.2
HIS	324	4 ± 3

**Table 3:** Root-mean-square deviation (RMSD), radius of gyration (RG), solvent-accessible surface area (SASA), hydrogen bonds (HB) and salt bridges (SB) averaged over the last 20 ns or in the last frame of MD simulations at different pHs.

pH	RMSD (nm) ${}^{a}$	RG (nm) ${}^{b}$	int-RG ${}^{c}$	ext-RG ${}^{d}$	SASA (nm${}^{2}$) ^*e*^	HB ${}^{f}$	SB ${}^{g}$	Exp. Activity ${}^{h}$
2	0.81 ± 0.02	6.56 ± 0.01	2.69 ± 0.02	8.34 ± 0.02	3589 ± 13	5853 ± 40	96	0
3	0.83 ± 0.05	6.60 ± 0.03	2.79 ± 0.04	8.40 ± 0.04	3482 ± 28	6044 ± 56	131	0
4	0.45 ± 0.01	6.29 ± 0.01	2.46 ± 0.01	8.10 ± 0.01	3182 ± 11	6518 ± 41	183	0
5	0.38 ± 0.01	6.24 ± 0.01	2.41 ± 0.01	8.07 ± 0.01	3052 ± 11	6973 ± 42	179	0.38
6	0.33 ± 0.01	6.20 ± 0.01	2.40 ± 0.01	8.02 ± 0.01	2893 ± 11	7287 ± 44	182	1.75
7	0.35 ± 0.01	6.22 ± 0.01	2.42 ± 0.01	8.03 ± 0.01	2852 ± 9	7549 ± 47	169	4.50
7.5	0.32 ± 0.01	6.18 ± 0.01	2.35 ± 0.01	8.01 ± 0.01	2828 ± 11	7460 ± 41	179	5.50

${}^{a}$ Root-mean-square deviation of the backbone atoms of the entire structure of urease, ${}^{b}$ radius of gyration of the entire urease, ${}^{c}$ radius of gyration of internal Glu505, ${}^{d}$ radius of gyration of external Glu101, Glu116 and Glu386, ^*e*^ solvent-accessible surface area of the entire urease, ${}^{f}$ total number of intramolecular hydrogen bonds of urease, ${}^{g}$ Intramolecular urease salt bridges in the last snapshot of the simulations, ${}^{h}$ experimental urease activity in μ mols/min/mg from reference [15].

## Supplementary Materials

The following are available online at https://www.mdpi.com/2073-4360/12/11/2713/s1, Figure S1: MD analysis of flap mobility at different pHs. Representation of the temporal evolution of the RMSD of the 12 flaps of urease enzyme at pH 2, 3, 4, 5, 6, 7 and 7.5, Figure S2: Representation of the temporal evolution of the RMSD values of each of the 12 urease dimers at different pHs, Table S1: Number of water molecules and ions used in the molecular dynamics simulations at different pHs, Table S2: Percentile scores of Sidechain and Ramachandran angles of residues of urease for crystal structure and for obtained structures after NVT and NPT simulations, List of force field parameters of the modified carbamylated lysine amino acid used in the Gromacs molecular dynamics simulations, and pKa values of the *H. pylori* urease residues obtained from Propka3 software.

## Abbreviations

The following abbreviations are used in this manuscript:

HB	hydrogen bond
HP	*Helicobacter pylori*
MD	molecular dynamics
QM	quantum mechanical
SASA	solvent-accessible surface area (SASA)
SB	salt bridge
SGCMC	semi-grand canonical Monte Carlo
RG	radius of gyration
RMSD	root-mean-square deviation

## References

[1] Ansari S., Yamaoka Y. (2017). Survival of Helicobacter pylori in gastric acidic territory. Helicobacter.

[2] Ha N.C., Oh S.T., Sung J.Y., Cha K.A., Lee M.H., Oh B.H. (2001). Supramolecular assembly and acid resistance of Helicobacter pylori urease. Nat. Struct. Biol..

[3] Schreiber S., Bücker R., Groll C., Azevedo-Vethacke M., Garten D., Scheid P., Friedrich S., Gatermann S., Josenhans C., Suerbaum S. (2005). Rapid loss of motility of Helicobacter pylori in the gastric lumen in vivo. Infect. Immun..

[4] Ottemann K.M., Lowenthal A.C. (2002). Helicobacter pylori uses motility for initial colonization and to attain robust infection. Infect. Immun..

[5] Kao C.Y., Sheu B.S., Wu J.J. (2016). Helicobacter pylori infection: An overview of bacterial virulence factors and pathogenesis. Biomed. J..

[6] Arora R., Issar U., Kakkar R. (2018). In Silico study of the active site of Helicobacter pylori urease and its inhibition by hydroxamic acids. J. Mol. Graph. Model..

[7] Estiu G., Merz K.M. (2006). Catalyzed decomposition of urea. Molecular dynamics simulations of the binding of urea to urease. Biochemistry.

[8] Roberts B.P., Miller B.R., Roitberg A.E., Merz K.M. (2012). Wide-open flaps are key to urease activity. J. Am. Chem. Soc..

[9] Minkara M.S., Ucisik M.N., Weaver M.N., Merz K.M. (2014). Molecular dynamics study of Helicobacter pylori urease. J. Chem. Theory Comput..

[10] Xu Y.P.P., Qin J., Sun S.M.M., Liu T.T.T., Zhang X.L.L., Qian S.S.S., Zhu H.L.L. (2014). Synthesis, crystal structures, molecular docking and urease inhibitory activity of nickel(II) complexes with 3-pyridinyl-4-amino-5-mercapto-1,2,4-triazole. Inorganica Chim. Acta.

[11] Macomber L., Minkara M.S., Hausinger R.P., Merz K.M. (2015). Reduction of urease activity by interaction with the flap covering the active site. J. Chem. Inf. Model..

[12] Sumner J.B. (1926). The isolation and crystallization of the enzyme urease preliminary paper. J. Biol. Chem..

[13] Anderson D.E., Becktel W.J., Dahlquist F.W. (1990). pH-induced denaturation of proteins: A single salt bridge contributes 3-5 kcal/mol to the free energy of folding of T4 lysozyme. Biochemistry.

[14] Bashford D., Karplus M. (1990). pKa’s of ionizable groups in proteins: Atomic detail from a continuum electrostatic model. Biochemistry.

[15] Scott D.R., Weeks D., Hong C., Postius S., Melchers K., Sachs G. (1998). The role of internal urease in acid resistance of Helicobacter pylori. Gastroenterology.

[16] Madurga S., Garcés J.L., Companys E., Rey-Castro C., Salvador J., Galceran J., Vilaseca E., Puy J., Mas F. (2009). Ion binding to polyelectrolytes: Monte Carlo simulations versus classical mean field theories. Theor. Chem. Accounts.

[17] Madurga S., Rey-Castro C., Pastor I., Vilaseca E., David C., Garcés J.L., Puy J., Mas F. (2011). A semi-grand canonical Monte Carlo simulation model for ion binding to ionizable surfaces: Proton binding of carboxylated latex particles as a case study. J. Chem. Phys..

[18] Blanco P.M., Madurga S., Mas F., Garcés J.L. (2018). Coupling of charge regulation and conformational equilibria in linearweak polyelectrolytes: Treatment of long-range interactions via effective short-ranged and pH-dependent interaction parameters. Polymers.

[19] Blanco P.M., Madurga S., Narambuena C.F., Mas F., Garcés J.L. (2019). Role of Charge Regulation and Fluctuations in the Conformational and Mechanical Properties of Weak Flexible Polyelectrolytes. Polymers.

[20] Olsson M.H., Søndergaard C.R., Rostkowski M., Jensen J.H. (2011). PROPKA3: Consistent treatment of internal and surface residues in empirical p K a predictions. J. Chem. Theory Comput..

[21] López A., Vilaseca M., Madurga S., Varese M., Tarragó T., Giralt E. (2016). Analyzing slowly exchanging protein conformations by ion mobility mass spectrometry: Study of the dynamic equilibrium of prolyl oligopeptidase. J. Mass Spectrom..

[22] Van Der Spoel D., Lindahl E., Hess B., Groenhof G., Mark A.E., Berendsen H.J. (2005). GROMACS: Fast, flexible, and free. J. Comput. Chem..

[23] Abraham M.J., Murtola T., Schulz R., Páll S., Smith J.C., Hess B., Lindah E. (2015). Gromacs: High performance molecular simulations through multi-level parallelism from laptops to supercomputers. SoftwareX.

[24] Horn H.W., Swope W.C., Pitera J.W., Madura J.D., Dick T.J., Hura G.L., Head-Gordon T. (2004). Development of an improved four-site water model for biomolecular simulations: TIP4P-Ew. J. Chem. Phys..

[25] West A.P., Millar M.R., Tompkins D.S. (1992). Effect of physical environment on survival of Helicobacter pylori. J. Clin. Pathol..

[26] Darden T., York D., Pedersen L. (1993). Particle mesh Ewald: An N log (N) method for Ewald sums in large systems. J. Chem. Phys..

[27] Bussi G., Donadio D., Parrinello M. (2007). Canonical sampling through velocity rescaling. J. Chem. Phys..

[28] Parrinello M., Rahman A. (1981). Polymorphic transitions in single crystals: A new molecular dynamics method. J. Appl. Phys..

[29] Humphrey W., Dalke A., Schulten K. (1996). VMD: Visual molecular dynamics. J. Mol. Graph..

[30] Pettersen E.F., Goddard T.D., Huang C.C., Couch G.S., Greenblatt D.M., Meng E.C., Ferrin T.E. (2004). UCSF Chimera—A visualization system for exploratory research and analysis. J. Comput. Chem..

[31] Dunn B.E., Campbell G.P., Perez-Perez G., Blaser M. (1990). Purification and characterization of urease from Helicobacter pylori. J. Biol. Chem..

[32] Turbett G.R., Høj P., Horne R., Mee B.J. (1992). Purification and characterization of the urease enzymes of Helicobacter species from humans and animals. Infect. Immun..

[33] Pinkse M.W.H., Maier C.S., Kim J.i., Oh B.h., Heck A.J.R. (2003). Macromolecular assembly of Helicobacter pylori urease investigated by mass spectrometry. J. Mass Spectrom..

[34] Scott D.R., Marcus E.A., Weeks D.L., Sachs G. (2002). Mechanisms of acid resistance due to the urease system of Helicobacter pylori. Gastroenterology.

