# Supplementary material for: Effect of pH on the Supramolecular Structure of Helicobacter pylori Urease by Molecular Dynamics Simulations
Source: Polymers (Basel). 2020 Nov 17;12(11):2713. doi: 10.3390/polym12112713 (PMC7696613; doi:10.3390/polym12112713)
Supplement: Supplementary file 1 [file polymers-12-02713-s001.pdf]

Supporting Information: Effect of pH on the supramolecular structure of Helicobacter pylori urease by molecular dynamics simulations.

Haruna L. Barazorda-Ccahuana,[†] Badhin Gómez,[‡] Francesc Mas,[†] and Sergio Madurga[*,†]

†*Materials Science and Physical Chemistry Department and IQTCUB, Universitat de Barcelona, Martí i Franquès, 1 - Barcelona, Spain*

‡*Centro de Investigación en Ingeniería Molecular - CIIM, Universidad Católica de Santa María, Urb. San José s/n - Umacollo, Arequipa, Perú*

E-mail: s.madurga@ub.edu

Table S1: Number of water molecules and ions used in the molecular dynamics simulations at different pHs

**pH**	**TIP4P Water**	**Ions**
2	148944	1373 Cl
3	149121	1208 Cl
4	149573	810 Cl
5	149987	438 Cl
6	150261	189 Cl
7	150465	3 Na
7.5	150391	83 Na

Table S2: Percentile scores of Sidechain and Ramachandran angles of residues of urease for crystal structure and for obtained structures after NVT and NPT simulations.

**Structural model**	**Poor rotamers**	**Favored rotamers**	**Ramachandran outliers**	**Ramachandran favored**
Crystal	9.90%	77.60%	7.10%	76.10%
After 10 ns NVT simulations				
pH2	6.6%	81.6%	3.0%	86.4%
pH3	6.9%	81.7%	2.8%	87.0%
pH4	6.5%	82.5%	2.6%	87.9%
pH5	6.6%	82.3%	2.7%	87.9%
pH6	6.4%	82.9%	2.6%	87.3%
pH7	6.3%	82.7%	2.5%	88.2%
pH7.5	6.5%	82.0%	2.6%	87.7%
After 100 ns NPT simulations				
pH2	4.2%	86.4%	1.9%	87.0%
pH3	3.9%	86.5%	2.3%	86.6%
pH4	4.2%	85.5%	2.1%	87.1%
pH5	4.1%	86.4%	2.1%	87.2%
pH6	4.8%	84.8%	2.0%	87.4%
pH7	4.9%	85.3%	2.1%	87.1%
pH7.5	4.6%	85.4%	1.8%	87.4%
After 200 ns NPT simulations				
pH2	3.7%	87.1%	2.0%	87.1%
pH7.5	4.4%	85.9%	2.0%	87.4%

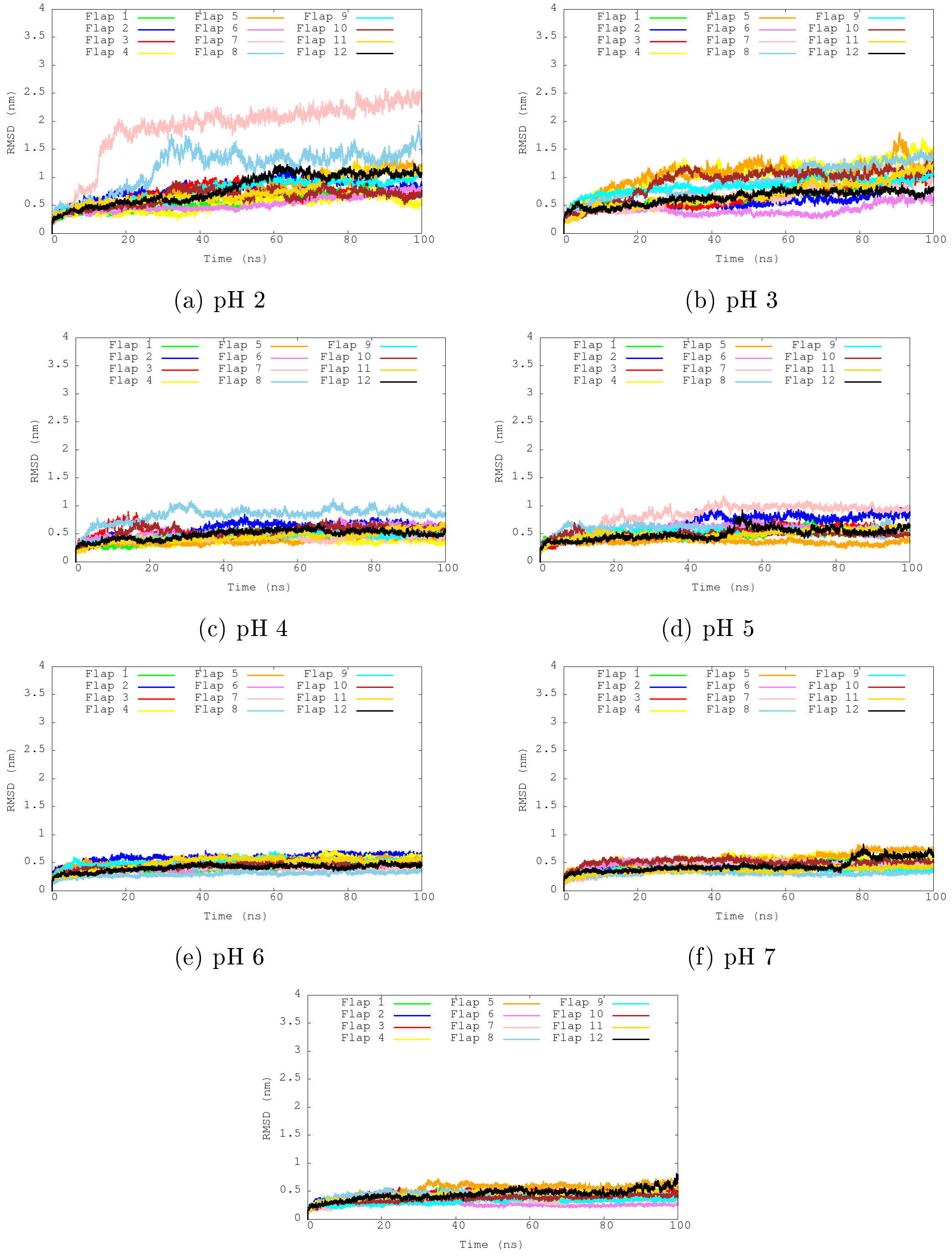

(a) pH 2 (b) pH 3

(c) pH 4 (d) pH 5

(e) pH 6 (f) pH 7

(g) pH 7.5

Figure S1: MD analysis of flap mobility at different pHs. Representation of the temporal evolution of the RMSD of the 12 flaps of urease enzyme at pH 2, 3, 4, 5, 6, 7 and 7.5.

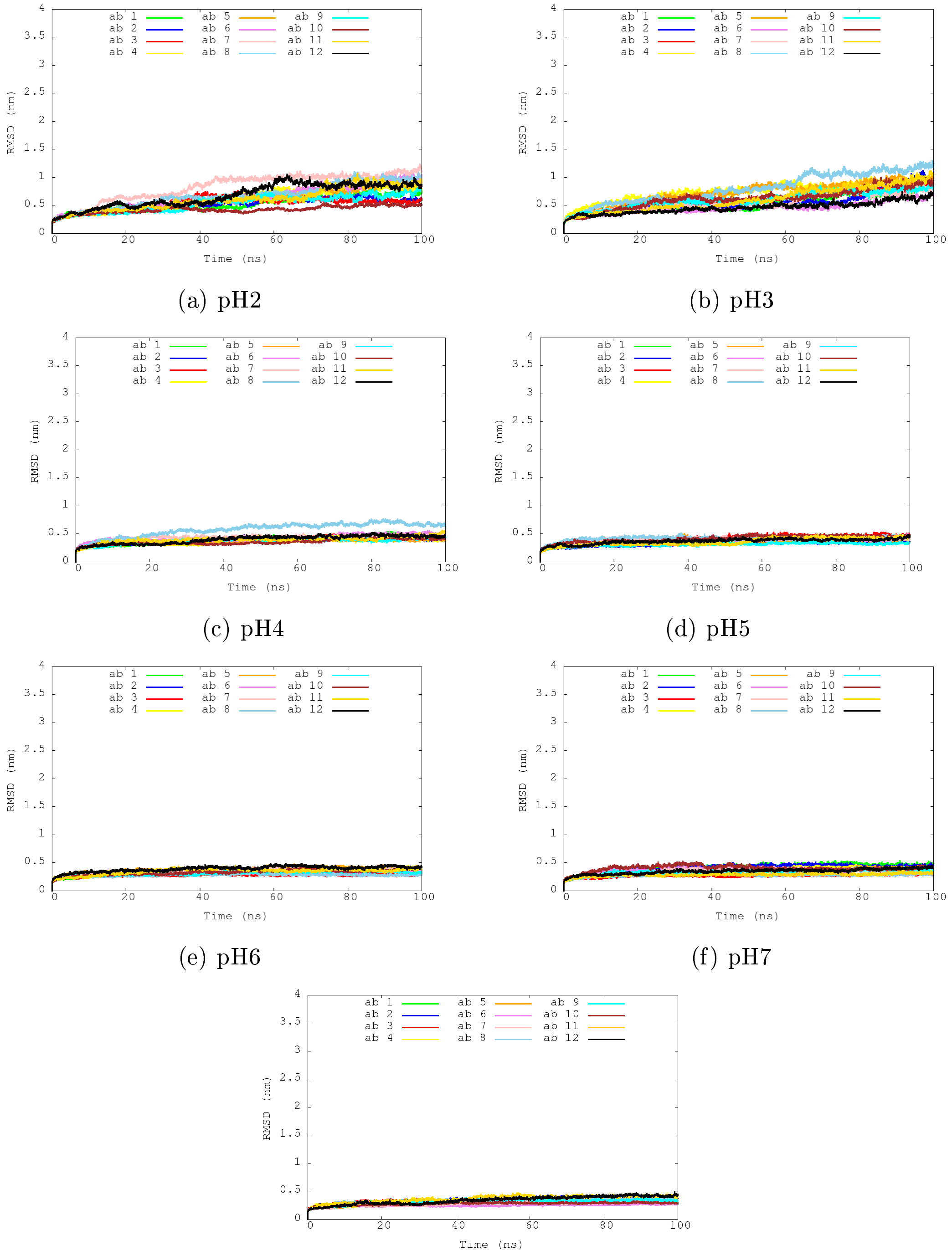

(a) pH2

(b) pH3

(c) pH4

(d) pH5

(e) pH6

(f) pH7

(g) pH7.5

Figure S2: Representation of the temporal evolution of the RMSD values of each of the 12 urease dimers at different pHs.

Forde field parameters

Force field parameters of the modified carbamylated lysine amino acid used in the Gromacs molecular dynamics simulations.

```
;OPLS-AA force field for KCX


[  KCX ]


[ atoms ]
  N      opls_238   -0.500     1
  H      opls_241    0.300     1
  CA  opls_224B   0.140     1
  HA  opls_140    0.060     1
  CB  opls_136   -0.120     2
  HB1  opls_140    0.060     2
  HB2  opls_140    0.060     2
  CG  opls_136   -0.120     3
  HG1  opls_140    0.060     3
  HG2  opls_140    0.060     3
  CD  opls_136   -0.120     4
  HD1  opls_140    0.060     4
  HD2  opls_140    0.060     4
  CE  opls_906    0.060     5
  HE1  opls_911    0.060     5
  HE2  opls_911    0.060     5
  NZ  opls_238   -0.500     6
  HZ1  opls_241    0.300     6
```

```
 C      opls_235    0.500     7
 O      opls_236   -0.500     7
 CX  opls_271    0.700     8
 OQ1  opls_272   -0.840     8
 OQ2  opls_272   -0.840     8

[ bonds ]
 OQ2    CX
 OQ1    CX
 HE1    CE
 HD2    CD
 CE     HE2
 HD1    CD
 HB2    CB
 CG     HG2
 CG     HG1
 HB1    CB
 CA     HA
 CX     NZ
 CE     NZ
 CA     N
 CE     CD
 CD     CG
 CG     CB
 CB     CA
 NZ     HZ1
 H      N
```

```
 CA     C
 O      C
 -C     N

[ angles ]
  NZ   CX  OQ2 dhTPP_NT_C_3_O2
  NZ   CX  OQ1 dhTPP_NT_C_3_O2

[ dihedrals ] ; override some with residue-specific ones
     N    CA    CB    CG    dih_LYS_chi1_N_C_C_C
    CG    CB    CA     C    dih_LYS_chi1_C_C_C_CO
    CD    CE    NZ   HZ1    dih_LYS_chi5_C_C_N_H
   OQ2    CX    NZ    CE    dhTPP_O2_C_3_NT_CT
   OQ1    CX    NZ    CE    dhTPP_O2_C_3_NT_CT
     N    CA     C     O    dhTPP_NT_CT_C_2_O_2

[ impropers ]
  NZ  OQ1   CX  OQ2 improper_O_C_X_Y
  -C   CA    N    H improper_Z_N_X_Y
  CA   +N    C    O improper_O_C_X_Y
```

```
;OPLS-AA force field for Niquel

[ NIK ]

 [ atoms ]
  NI1  opls_966   2.000     0
  NI2  opls_966   2.000     0

 [ bonds ]
    NI1  NI2

;OPLS-AA force field for OH

[ OH ]

 [ atoms ]
    OW   opls_116   -1.20      0
   HW1   opls_117    0.20      0

 [ bonds ]
    OW   HW1
```

pKa values of the *H. pylori* urease residues obtained from Propka3 software.

Group			pKa	model-pKa
ASP	9	A	4.41	3.80
ASP	68	A	3.96	3.80
ASP	69	A	3.21	3.80
ASP	72	A	3.99	3.80
ASP	89	A	3.52	3.80
ASP	117	A	3.98	3.80
ASP	136	A	4.49	3.80
ASP	155	A	4.14	3.80
ASP	157	A	3.50	3.80
ASP	167	A	3.63	3.80
ASP	188	A	2.82	3.80
ASP	203	A	5.05	3.80
ASP	207	A	4.09	3.80
ASP	229	A	3.10	3.80
ASP	230	A	3.43	3.80
GLU	7	A	4.59	4.50
GLU	18	A	4.35	4.50
GLU	25	A	4.00	4.50
GLU	34	A	2.96	4.50
GLU	45	A	2.51	4.50
GLU	46	A	5.22	4.50
GLU	56	A	4.09	4.50
GLU	60	A	3.62	4.50

GLU	80	A	4.43	4.50
GLU	84	A	4.64	4.50
GLU	101	A	4.52	4.50
GLU	110	A	4.99	4.50
GLU	116	A	4.26	4.50
GLU	122	A	4.13	4.50
GLU	149	A	8.95	4.50
GLU	159	A	4.11	4.50
GLU	177	A	4.57	4.50
GLU	180	A	3.76	4.50
GLU	181	A	3.46	4.50
GLU	185	A	3.95	4.50
GLU	209	A	3.66	4.50
GLU	220	A	3.98	4.50
GLU	238	A	2.42	4.50
C-	238	A	2.10	3.20
HIS	14	A	3.02	6.50
HIS	42	A	5.76	6.50
HIS	79	A	6.43	6.50
HIS	97	A	6.73	6.50
HIS	144	A	3.41	6.50
HIS	146	A	2.74	6.50
HIS	216	A	7.40	6.50
HIS	224	A	6.37	6.50
CYS	153	A	10.69	9.00
TYR	15	A	16.40	10.00
TYR	32	A	12.58	10.00

TYR	232	A	12.01	10.00
LYS	2	A	7.01	10.50
LYS	6	A	12.19	10.50
LYS	10	A	10.29	10.50
LYS	21	A	10.86	10.50
LYS	22	A	8.90	10.50
LYS	24	A	9.82	10.50
LYS	26	A	10.63	10.50
LYS	29	A	10.18	10.50
LYS	51	A	10.27	10.50
LYS	52	A	11.78	10.50
LYS	66	A	11.02	10.50
LYS	92	A	8.34	10.50
LYS	105	A	10.50	10.50
LYS	114	A	10.60	10.50
LYS	124	A	9.01	10.50
LYS	125	A	9.86	10.50
LYS	130	A	11.48	10.50
LYS	132	A	10.51	10.50
LYS	160	A	11.59	10.50
LYS	164	A	10.24	10.50
LYS	182	A	10.53	10.50
LYS	211	A	10.70	10.50
LYS	212	A	11.34	10.50
LYS	219	A	10.33	10.50
LYS	227	A	10.57	10.50
LYS	234	A	10.76	10.50

LYS	237	A	10.21	10.50
ARG	23	A	14.03	12.50
ARG	48	A	12.32	12.50
ARG	62	A	12.37	12.50
ARG	137	A	15.83	12.50
ARG	152	A	13.27	12.50
ARG	158	A	8.81	12.50
ARG	165	A	12.20	12.50
ARG	175	A	11.59	12.50
ARG	193	A	11.64	12.50
ARG	194	A	12.46	12.50
ARG	204	A	11.96	12.50
ARG	217	A	12.72	12.50
ARG	221	A	11.96	12.50
N+	1	A	3.03	8.00
ASP	257	B	5.14	3.80
ASP	263	B	6.41	3.80
ASP	265	B	3.40	3.80
ASP	273	B	4.97	3.80
ASP	305	B	6.04	3.80
ASP	315	B	6.12	3.80
ASP	323	B	6.64	3.80
ASP	328	B	3.86	3.80
ASP	341	B	5.61	3.80
ASP	344	B	3.58	3.80
ASP	372	B	7.14	3.80
ASP	403	B	4.05	3.80

ASP	442	B	4.10	3.80
ASP	447	B	4.30	3.80
ASP	461	B	7.98	3.80
ASP	474	B	3.58	3.80
ASP	477	B	4.44	3.80
ASP	480	B	3.92	3.80
ASP	488	B	8.54	3.80
ASP	498	B	5.29	3.80
ASP	523	B	3.16	3.80
ASP	554	B	4.90	3.80
ASP	563	B	4.02	3.80
ASP	569	B	5.91	3.80
ASP	574	B	4.24	3.80
ASP	586	B	7.98	3.80
ASP	590	B	4.67	3.80
ASP	600	B	10.36	3.80
ASP	619	B	2.78	3.80
ASP	634	B	3.38	3.80
ASP	636	B	4.51	3.80
ASP	671	B	4.64	3.80
ASP	700	B	6.52	3.80
ASP	726	B	3.67	3.80
ASP	738	B	3.94	3.80
ASP	764	B	4.06	3.80
ASP	786	B	4.05	3.80
GLU	246	B	4.52	4.50
GLU	269	B	5.57	4.50

GLU	271	B	3.42	4.50
GLU	279	B	2.68	4.50
GLU	280	B	7.15	4.50
GLU	291	B	6.50	4.50
GLU	302	B	4.70	4.50
GLU	303	B	6.46	4.50
GLU	357	B	6.82	4.50
GLU	362	B	1.59	4.50
GLU	425	B	4.47	4.50
GLU	426	B	2.74	4.50
GLU	450	B	4.03	4.50
GLU	460	B	8.53	4.50
GLU	492	B	5.92	4.50
GLU	497	B	3.74	4.50
GLU	514	B	6.61	4.50
GLU	530	B	4.84	4.50
GLU	549	B	5.22	4.50
GLU	551	B	4.19	4.50
GLU	568	B	5.23	4.50
GLU	585	B	10.99	4.50
GLU	609	B	2.54	4.50
GLU	624	B	3.72	4.50
GLU	630	B	3.15	4.50
GLU	631	B	7.26	4.50
GLU	659	B	4.26	4.50
GLU	665	B	3.47	4.50
GLU	715	B	3.79	4.50

GLU	743	B	4.13	4.50
GLU	744	B	4.28	4.50
GLU	748	B	3.86	4.50
GLU	775	B	4.27	4.50
GLU	779	B	4.64	4.50
GLU	789	B	5.92	4.50
C-	807	B	3.17	3.20
HIS	272	B	6.27	6.50
HIS	374	B	-2.00	6.50
HIS	376	B	3.00	6.50
HIS	459	B	-0.21	6.50
HIS	471	B	6.75	6.50
HIS	486	B	-4.46	6.50
HIS	509	B	1.72	6.50
HIS	512	B	1.30	6.50
HIS	520	B	3.76	6.50
HIS	531	B	6.21	6.50
HIS	552	B	2.77	6.50
HIS	560	B	1.91	6.50
HIS	561	B	5.65	6.50
HIS	589	B	1.03	6.50
HIS	655	B	3.30	6.50
HIS	719	B	6.35	6.50
HIS	720	B	3.44	6.50
HIS	773	B	6.76	6.50
HIS	782	B	6.61	6.50
CYS	495	B	12.64	9.00

CYS	559	B	13.26	9.00
CYS	757	B	12.64	9.00
TYR	247	B	19.13	10.00
TYR	251	B	16.45	10.00
TYR	274	B	10.29	10.00
TYR	277	B	11.88	10.00
TYR	316	B	13.35	10.00
TYR	320	B	12.91	10.00
TYR	427	B	15.98	10.00
TYR	479	B	12.32	10.00
TYR	643	B	16.38	10.00
TYR	647	B	13.31	10.00
TYR	660	B	12.67	10.00
TYR	712	B	13.90	10.00
TYR	713	B	14.77	10.00
TYR	725	B	15.83	10.00
TYR	737	B	10.36	10.00
TYR	781	B	10.72	10.00
LYS	240	B	9.69	10.50
LYS	241	B	10.51	10.50
LYS	245	B	10.48	10.50
LYS	258	B	10.50	10.50
LYS	282	B	10.96	10.50
LYS	287	B	8.08	10.50
LYS	301	B	10.45	10.50
LYS	321	B	9.00	10.50
LYS	327	B	10.56	10.50

LYS	330	B	9.91	10.50
LYS	336	B	9.98	10.50
LYS	340	B	10.25	10.50
LYS	347	B	7.98	10.50
LYS	418	B	9.40	10.50
LYS	436	B	6.60	10.50
LYS	478	B	10.78	10.50
LYS	526	B	10.48	10.50
LYS	564	B	9.78	10.50
LYS	567	B	10.54	10.50
LYS	620	B	9.18	10.50
LYS	622	B	10.22	10.50
LYS	623	B	10.77	10.50
LYS	629	B	11.38	10.50
LYS	632	B	10.43	10.50
LYS	641	B	10.24	10.50
LYS	646	B	5.42	10.50
LYS	668	B	9.14	10.50
LYS	683	B	8.92	10.50
LYS	689	B	7.92	10.50
LYS	722	B	11.31	10.50
LYS	724	B	9.60	10.50
LYS	739	B	10.02	10.50
LYS	742	B	9.87	10.50
LYS	755	B	10.79	10.50
LYS	762	B	7.28	10.50
LYS	763	B	10.36	10.50

LYS	788	B	10.24	10.50
LYS	793	B	11.38	10.50
LYS	797	B	9.81	10.50
ARG	244	B	12.75	12.50
ARG	260	B	12.41	12.50
ARG	290	B	12.92	12.50
ARG	414	B	12.51	12.50
ARG	415	B	11.60	12.50
ARG	422	B	13.12	12.50
ARG	506	B	11.54	12.50
ARG	576	B	11.24	12.50
ARG	578	B	10.49	12.50
ARG	606	B	16.57	12.50
ARG	613	B	11.68	12.50
ARG	627	B	12.65	12.50
ARG	639	B	13.61	12.50
ARG	642	B	11.13	12.50
ARG	714	B	13.98	12.50
ARG	749	B	9.71	12.50
ARG	758	B	11.10	12.50
N+	239	B	8.37	8.00
